# Microtubule acetylation dyshomeostasis in Parkinson’s disease

**DOI:** 10.1186/s40035-023-00354-0

**Published:** 2023-05-08

**Authors:** Padmashri Naren, Khan Sabiya Samim, Kamatham Pushpa Tryphena, Lalitkumar K. Vora, Saurabh Srivastava, Shashi Bala Singh, Dharmendra Kumar Khatri

**Affiliations:** 1 Molecular and Cellular Neuroscience Lab, Department of Pharmacology and Toxicology, National Institute of Pharmaceutical Education and Research (NIPER), Hyderabad, Telangana 500037 India; 2 School of Pharmacy, Queen’s University Belfast, 97 Lisburn Road, Belfast, BT9 7BL UK; 3 Department of Pharmaceutics, National Institute of Pharmaceutical Education and Research (NIPER), Hyderabad, Telangana 500037 India

**Keywords:** Parkinson’s disease, Microtubule acetylation, Tubulin, PTMs, Mitochondria, Axonal transport, HDAC6, SIRT2 inhibitors

## Abstract

**Abstract:**

The inter-neuronal communication occurring in extensively branched neuronal cells is achieved primarily through the microtubule (MT)-mediated axonal transport system. This mechanistically regulated system delivers cargos (proteins, mRNAs and organelles such as mitochondria) back and forth from the soma to the synapse. Motor proteins like kinesins and dynein mechanistically regulate polarized anterograde (from the soma to the synapse) and retrograde (from the synapse to the soma) commute of the cargos, respectively. Proficient axonal transport of such cargos is achieved by altering the microtubule stability via post-translational modifications (PTMs) of α- and β-tubulin heterodimers, core components constructing the MTs. Occurring within the lumen of MTs, K40 acetylation of α-tubulin via α-tubulin acetyl transferase and its subsequent deacetylation by HDAC6 and SIRT2 are widely scrutinized PTMs that make the MTs highly flexible, which in turn promotes their lifespan. The movement of various motor proteins, including kinesin-1 (responsible for axonal mitochondrial commute), is enhanced by this PTM, and dyshomeostasis of neuronal MT acetylation has been observed in a variety of neurodegenerative conditions, including Alzheimer’s disease and Parkinson’s disease (PD). PD is the second most common neurodegenerative condition and is closely associated with impaired MT dynamics and deregulated tubulin acetylation levels. Although the relationship between status of MT acetylation and progression of PD pathogenesis has become a chicken-and-egg question, our review aims to provide insights into the MT-mediated axonal commute of mitochondria and dyshomeostasis of MT acetylation in PD. The enzymatic regulators of MT acetylation along with their synthetic modulators have also been briefly explored. Moving towards a tubulin-based therapy that enhances MT acetylation could serve as a disease-modifying treatment in neurological conditions that lack it.

**Graphical abstract:**

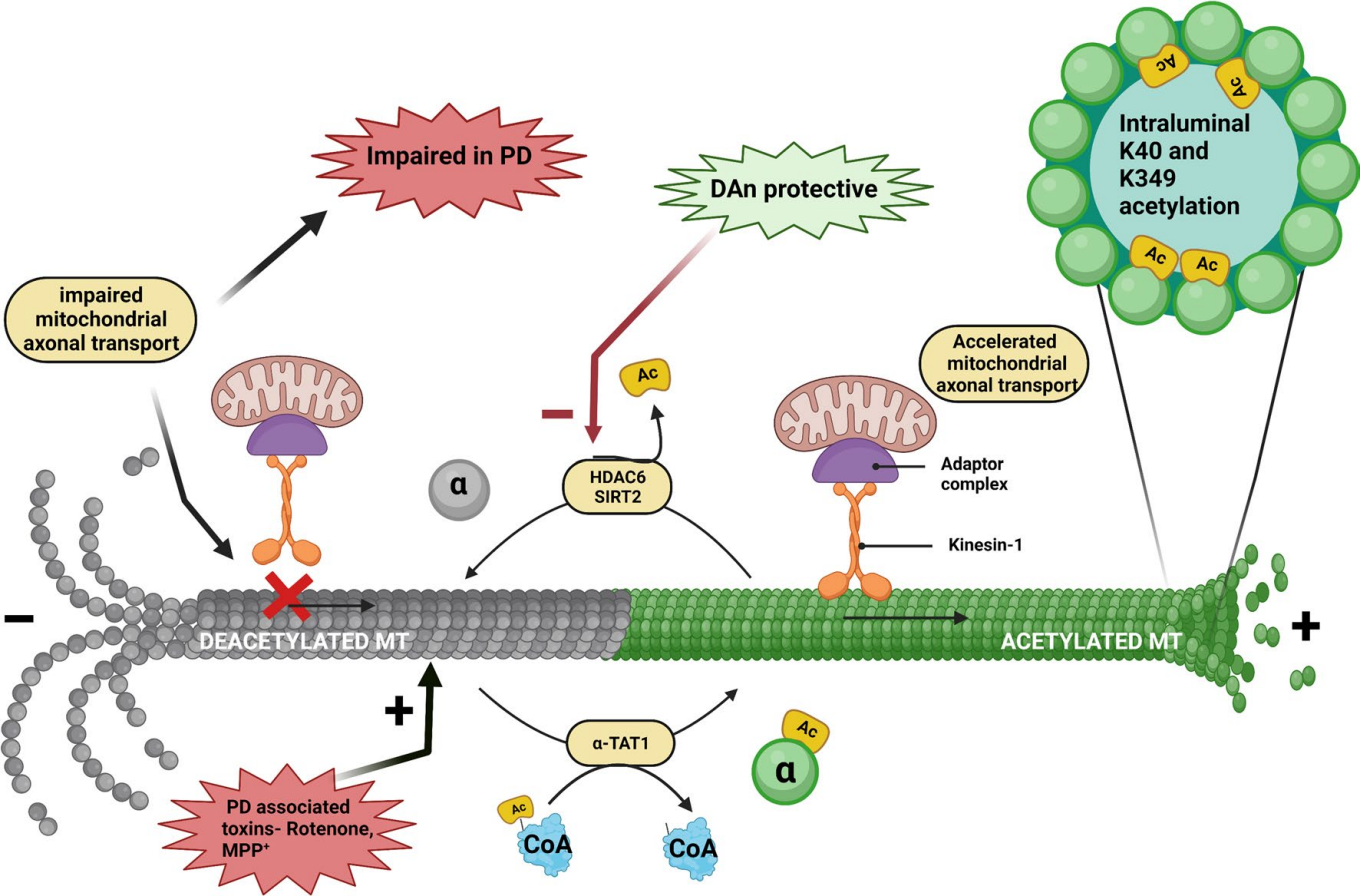

## Background

Inside the brain, there exists a dynamic network formed by billions of nerve cells that process information in the form of electrical signals. Each neuron receives such signals from thousands of other neurons through multiple processes, called dendrites, and reports them to the subsequent neuron via a single axon. The arrival of these electrical signals at the synapse triggers the regulated release of various neurotransmitters that help in communication between neurons by further signal transmission. Apart from such interneuronal communications, there are highly complex and brimming intraneuronal communications occurring within a single neuron on a daily basis [1]. The dendrites and the axon are in constant correspondence with one another through the help of intraneuronal scaffolding proteins. However, how does a presynaptic cargo such as an organelle, vesicle, protein, lipid and synaptic components made at the soma, end up at an appropriate place within a neuron? The microtubule (MT) cytoskeleton is responsible for cargo transport inside the nerve cell and governs the polarized delivery and removal of cargos over long distances in addition to performing other intricate basal cellular functions, such as mitotic spindle formation during cell division, axoneme formation aiding in cellular motility and cellular cytoskeleton maintenance [2]. Parkinson’s disease (PD) is a well-elaborated neurodegenerative disorder that has been linked to the dysregulation of MT dynamics with findings of Lewy body-associated tubulins and neurofilaments in post-mortem PD brains. Similarly, PD-linked proteins such as LRRK2, PINK, Parkin, and α-synuclein (α-syn) have been shown to have an impact on microtubule stability. While PD is multifactorial [3–6], recent studies on PD pathogenesis have converged at MT dynamics [7, 8]. The integrity of the MT network is essential for the functional well-being of any cell, especially highly complex neurons. In addition to different expression levels of tubulin isotypes for functional variability, a governing regulatory mechanism for the strict control of MT dynamics and stability is the post-translational modifications (PTMs) of their functional protein unit, i.e., heterodimers of α- and β-tubulin [9]. Microtubule-associated proteins (MAPs) play a role in linking post-translational functional modifications such as acetylation, tyrosination, polyamination, SUMOylation, phosphorylation, palmitoylation, S-nitrosylation, ubiquitylation, glycosylation, succination and methylation to the dysfunctional status of tubulin proteins [9]. The imbalance of lysine acetylation/deacetylation in histone and non-histone proteins is closely connected with the pathogenesis of various diseases from cancer to neurodegeneration. Acetylation of tubulin plays an important role in regulating MT dynamics and stability. Tubulin acetylation is strongly associated with long-lived MTs and has consistently been proven to protect them from mechanical aging [10]. Acetylation-mediated enhanced flexibility of MTs renders them resistant to mechanical stress and lattice damage. Moreover, studies have shown that acetylated MTs form a better and efficient transport system for intraneuronal cargo transport. Genetic studies showed that knockdown or knockout of enzymes responsible for MT acetylation, such as α-tubulin acetyl transferase (αTAT-1), results in abnormal axonal growth and overbranching, indicating its importance for neuronal modeling [11]. MT-mediated intraneuronal transport of vital organelles has been observed to be relatively higher in acetylated MTs [12, 13]. With the discovery of a highly characteristic and handy antibody, studies conducted with genetic or pharmacological alterations of tubulin acetylation have made it possible to investigate the contributions of MT acetylationto neuronal health. Acetylation of α-tubulin has received much attention as an essential governing factor of microtubule dynamics. Impaired acetylation is closely related to several neurodegenerative conditions. Here, we will review the importance of acetylated MTs and their dysregulation in PD, along with the enzymatic modulators and their synthetic ligands regulating α-tubulin acetylation (see graphical abstract for illustration).

## Neuronal microtubules: structural overview and assembly

**Fig. 1:**
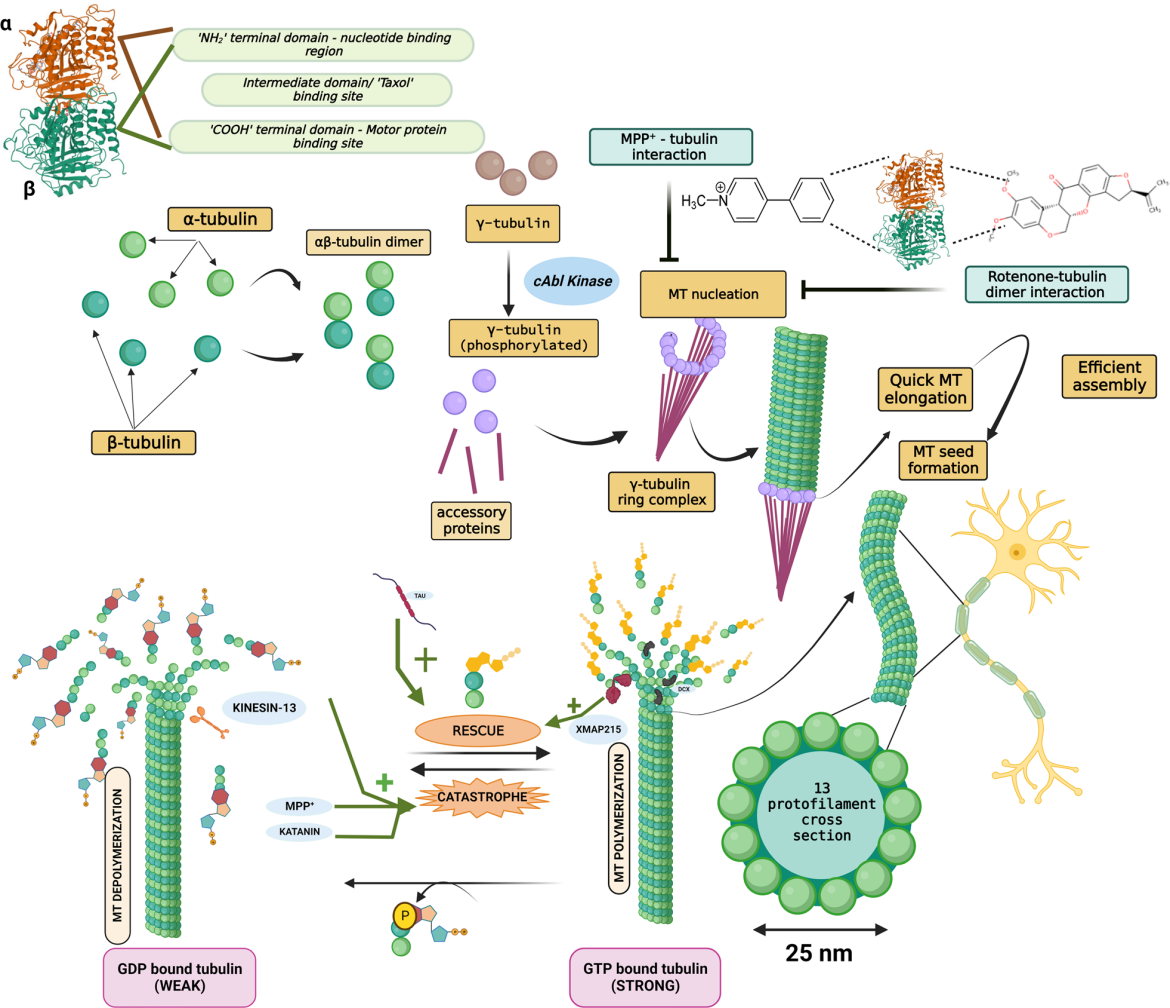
Microtubule (MT) assembly and dynamic instability. Each tubulin has three characteristic domains, and α-tubulin and β-tubulin form a heterodimer in a 1:1 stoichiometry. The γ-tubulin ring complex, formed by the association of phosphorylated γ-tubulin and accessory proteins, provides a base onto which the tubulin dimers are added spirally, leading to its quick elongation via polymerization. Completely formed MTs experience ‘dynamic instability’ characterized by routine, balanced cycles of rescue (stable GTP-bound β-tubulin remains intact and hence dimers are added) and catastrophe (GTP hydrolysis of β-tubulin results in its subsequent dissociation from the MTs and renders it unstable). XMAP215, tau and doublecortin  promote MT rescue, whereas katanin and kinesin-13 promote catastrophe. Additionally, the PD model toxins rotenone and MPP^+^ also destabilize MTs and orchestrate their fateful demise via catastrophe, collapsing the MT-network-associated axonal commute of vital substances

At the molecular level, microtubules are non-covalent cytoskeletal filaments composed of two different tubulin monomers, α-tubulin and β-tubulin, at a 1:1 ratio by stoichiometry. In vitro self-assembly of microtubules occurs when these monomers couple to form a dimer. A rate-limiting step called nucleation drives the lateral assembly of several dimers to form a hollow structure called the microtubule seed, upon which further stacking of the dimers occurs. In most cell types, this base template for filament extension is provided by the γ-tubulin ring complex (γTuRC), formed with the help of another protein, γ-tubulin [14]. Recent studies showed that γTuRC is initiated via c-Abl kinase-mediated phosphorylation of γ-tubulin at the Y443 residue, a process highly essential for γTuRC assembly and MT nucleation [15]. This alignment occurs such that all the α-tubulin tails are present toward the slowly building ‘minus’ end and the β-tubulin heads face the quickly forming ‘plus’ end. The αβ dimers assemble in a spiral form that layers on top of the seed/ring, and each subsequently added dimer lengthens the spiral until a full-fledged long, hollow tube is created. In parallel, the β-tubulin heads and α-tubulin tails are also associated in linear columns and polymerize in a GTP-dependent manner into protofilaments. The presence of a non-hydrolyzed GTP cap at the plus-end stabilizes it and dictates its efficient assembly. Microtubules generally have 13 protofilaments surrounding a rigid hollow core, and such 13-fold symmetry generates a wide tubular structure of 15–25 nm in diameter [16]. In addition to being intracellular communication highways, MTs are also involved in nucleic and cell division (chromosome segregation) and offer a unique architecture to neurons to maintain their exaggerated structure so that they will not collapse. For the accomplishment of such varied functions, constant remodeling of individual MTs is needed. They tend to undergo fluctuating polymerization and depolymerization phases that exhibit stochastic growth (called microtubule rescue) and rapid shrinking (called microtubule catastrophe), respectively [17]. This constant shuffling between the elongating and falling-apart states is termed ‘dynamic instability’ and is an inherent property of MTs. It is regulated by the rate of addition of tubulin dimers, which is in turn governed by MAPs. For instance, XMAP215 binds to the ‘plus’ end of microtubules to catalyze addition and promote rescue, whereas kinesin 13 destabilizes the ‘plus’ end, causing catastrophe [18, 19]. Figure 1 illustrates the chronological assembly of a MT along with its phases of rescue and catastrophe. Tau protein is another widely known MAP and is associated with several neurodegenerative disorders, including Alzheimer's disease (AD) and PD. Tau helps in MT bundling and promotes its growth and inhibits its shrinkage by regulating the labile domain of MT [20]. A recent study by Mecak et al. [21] proved the involvement of kinesin-14 motor proteins in regulating the dynamic instability of MTs. The MT cytoskeleton is constantly exposed to external mechanical forces arising from active ongoing processes within or outside the cell. Interactions of molecular motors (such as kinesins and dynein), severing proteins (such as katanins and spastins) and MAPs with MTs exert internal mechanical forces on them. The stability of MTs in the presence of these forces will dictate their integrity [22, 23]. Tubulin subunits are pulled out of the MT by the mechanical forces on the C-terminal tail exerted by MT-severing proteins. The force required to remove tubulin dimers from the ends of MTs is not well established, and the force required for their removal from the walls of MTs has remained elusive. But recently, single-molecule techniques have revealed that tens of piconewtons of mechanical force could potentially pull out the tubulin subunits from the MT lattice within seconds [24].

The neuronal cells have a distinct structural architecture characterized by long axons and intensely branching dendrites that sets them unique from other cell types. Such a striking morphology poses an evolutionary yearning for abundant energy distribution for its homeostatic regulation and maintenance. Synapses are the major site of ATP consumption, in which approximately 93% of the energy demand is met by the mitochondria, while the remaining 7% is produced by glycolysis [25]. Such a high energy demand requires sustained maintenance of energy throughout the cell, especially at the axon initial segment, nodes of Ranvier, growth cones and distal axonal terminals. This is achieved by constant shuttling of mitochondria across the soma and distal axon and dendritic terminals via a MT-based motor protein-dependent transport mechanism, which typically involves the motor proteins kinesin-1 and dynein. Impairment of such transport mechanisms has been reported in the pathogenesis of several neurodegenerative disorders, including AD, PD, Huntington’s disease (HD), and amyotrophic lateral sclerosis (ALS) [26]. Particularly, dopaminergic neurons of the substantia nigra pars compacta (SNpc) have a high energy demand owing to their inherent lack of myelination. Hence, axonal transport of energy-replenishing mitochondria is critical in this respect, and its malfunctioning is highly likely to play a role in the pathogenesis of PD [27].

## MT-mediated axonal trafficking

Axonal transport is a fundamental delivery system governing the commute of cargos such as organelles, regulatory and functional proteins, lipids, vesicles, mRNAs and other functional requisites necessary for healthy neuronal thriving. Axonal transport can be broadly classified into two categories—fast and slow axonal transport. Slow axonal transport has a velocity typically ranging 0.02–0.04 µm/s and is achieved by actin-based myosin motors, while fast axonal transport is purely MT-based with velocity ranging 0.1–0.7 µm/s [28]. Slow axonal transport is typical for cytosolic proteins, tubulin, neurofilaments, and RNA, while MT-based fast transport includes the signature anterograde and retrograde systems. Synaptic vesicles, amyloid protein precursor (APP) and brain-derived neurotrophic factor (BDNF) vesicles undergo anterograde transport, whereas signaling vesicles and autophagosomes undergo retrograde transport. In general, cellular vesicles mainly undergo fast axonal transport and the glycolytic machinery constantly provides energy for this commute [29]. Cytoplasmic organelles such as mitochondria, endosomes and lysosomes display bidirectional motility [30]. Highly polarized neurons, with their complex axon extending over hundreds of centimeters in length, demand extraordinarily high amounts of energy. Hence, constant mitochondrial commute within a neuron is cardinal to meet the local energy demand and its overall survival [31]. The mitochondria undergo long-distance bidirectional transport along the microtubule cytoskeleton toward the plus end (anterograde transport) as well as the minus end (retrograde transport). This is facilitated by two opposing motor proteins that use ATP hydrolysis as their driving force: plus end-directing kinesins and minus end-directing dynein, which drive mitochondrial transport from the soma to the synapse and vice versa, respectively. Apart from this, the organelle also exhibits periods of stationary docking. The ATP gradient generated by the organelle modulates intermitochondrial signaling and thereby modulates its motility and uniform axonal distribution [32].

### Anterograde signaling

The mechanistically regulated commute of the aforementioned cargos toward the synapse from the soma with the help of motor adaptor proteins is termed anterograde signaling. Major motor proteins involved in anterograde cargo transport include the kinesin family proteins. All the kinesin superfamily proteins (KIFs) involved in mammalian axonal transport are phylogenetically classified into subfamilies ranging from kinesin-1 to kinesin-14 (Kinesin-1, 2, 3, 4/10, 6, 8, 9, 13, 14, 16, 17, 18, 19 and 20) and are encoded by at least 45 different KIF motor genes. Additionally, each of these kinesin families has a wide taxonomic distribution among all eukaryotes [33]. Members of the kinesin-1 family (pooled under the term ‘KIF5’) play a crucial role in the plus end-directed anterograde transport of neuronal mitochondria, lysosomes, endosomes and APP. KIF5A, KIF5B and KIF5C are the three mammalian motor isoforms, among which KIF5A and KIF5C are neuron-specific, while KIF5B is ubiquitously expressed [26]. Structurally, kinesin-1 is a tetramer possessing two heavy chains and two light chains. Heavy chains form the motor and stalk domains, while light chains serve as regulators and cargo attachment sites. The amino terminal domain of each heavy chain has ATPase activity and binds to MTs, while the cargo proteins affix with the carboxy terminal of the light chains [34]. In vitro motility assays have reported the possibility that a two-headed kinesin-1 moiety moves along two parallel MT protofilaments at a velocity of 0.8 µm/s. These heads that are linked at the neck region have a size less than 10 nm but take remarkably large steps of 8 nm (spacing between successive tubulin dimers) within every 10 ms. The association and dissociation of the heads occur alternatively; for instance, when both ADP-bound heads interact with MTs, one head binds to the MT and rapidly dissociates its ADP, leaving the other head dissociated from its bound ADP. Such repetitive binding and hydrolysis of MT-associated ADP on the head steers a conformational change that aids in achieving stepwise right-left movement of kinesin-1 on the tubules [35]. The next thing to ponder would be, how in actuality do the kinesin-1 motor proteins attach to the mitochondria and regulate its polarized plus end-directed transport across the neuron? The recruitment of these molecular motors to the mitochondria is facilitated by the adaptor proteins trafficking kinesin-binding protein 1 and 2 (TRAK1 and TRAK2), which are mammalian orthologs of the protein Milton found in *D. melanogaster.* TRAK1 is densely populated in the axons, aids in axonal localization of mitochondria and has affinity for both kinesin-1 and dynein motors, while TRAK2 is found primarily in the dendrites and regulates the dendritic distribution of mitochondria by primarily interacting with only dynein moieties. However, endogenous KIF5 has a higher affinity to TRAK1 over TRAK2, implying that kinesin-1 could have weak interactions with the latter [36]. However, there has been a study reporting a contrasting finding of higher affinity of KIF5 to TRAK2 to mediate anterograde transport upon forming a complex with KIF5, dynein and dynactin [37]. These TRAK proteins in turn bind the mitochondrial outer membrane protein Miro, an RHO family GTPase. There are two Miro GTPases in mammals, namely, Miro1 and Miro2, encoded by genes Ras homolog family member T1 and T2 (*RHOT1 *and *RHOT2*) dwelling on chromosome 17, respectively. Miro contains two EF hand motifs that bind calcium ions and two structurally and functionally different GTPase domains (at both the C- and the N-termini), making it the only known human protein having two different GTPase domains. Miro is essentially involved in connecting TRAKs and KIF5 to the mitochondria [38]. Deacetylation of Miro1 at the K105 residue by the histone deacetylase (HDAC) 6 enzyme significantly impairs mitochondrial axonal transport, and inhibition of HDAC6 restores the commute [38]. Syntabulin is another widely recognized KIF5 motor adaptor protein for mitochondrial transport. Malfunctioning of syntabulin or impaired syntaphilin–KIF5 interaction results in a decline in the anterograde transport of mitochondria without much alteration of retrograde signaling [39]. Syntaphilin is a mitochondrial membrane protein that aids in tethering mitochondria to the MT and thereby halts mitochondrial transport. Syntaphilin A- and B-mediated mitochondrial docking in growth cones is important for the elongation of axons in vivo [40]*.*

Genetic data from a very recent study performed in *C. elegans* suggest metaxins MTX1 and MTX2 as crucial coupling motor proteins for mitochondrial transport in addition to TRAK and Miro. Metaxins are outer mitochondrial membrane proteins that normally form a constituent of the mitochondrial protein translation apparatus. MTX1 and MTX2 form a complex and bind to Miro and the tetracotripeptide repeat motif of kinesin light chain KLC-1. This tri-protein complex (Miro/metaxins) functions as an essential adaptor protein that couples mitochondria to kinesin-1 [41]. Apart from metaxins, *O*-linked β-*N*-acetylglucosamine (*O*-GlcNAc) transferase has also been found to interact with TRAK1/2, Miro and KIF5C to form a quarternary complex and assist in the arrest of mitochondrial motility [42]. The activity of Milton/TRAK1/2 is modified by glucose-mediated *O*-GlcNAcylation (O-linked β-*N*-acetylglucosamylation), signifying the integral role of glucose in altering mitochondrial motility [43]. In general, any impairment in the KIF5-mitochondria interaction will rapidly disrupt the anterograde movement of mitochondria and decrease the amount of mitochondria in neuronal end terminals. Miro1 has direct interactions with certain proteins encoded by PD-linked genes such as PTEN-induced kinase 1 (*PINK*1), *PARKIN* and Leucine-rich repeat kinase 2 (*LRRK*2) (as witnessed in human neuroblastoma cells and *D. melanogaster* primary neurons). Exome sequencing of four PD patients revealed heterozygous mutations in the *RHOT*1 gene. *RHOT*2 has been recently identified as a PD-associated gene by gene-based association clustering methods [44, 45]. Ideally, any malfunctioning mitochondria would be directed toward mitophagy, which requires their detachment from the microtubule cytoskeleton, thereby halting its axonal transport. Damaged mitochondria automatically recruit PINK1 due to the drop in membrane potential. Subsequent recruitment and phosphorylation of PARKIN by PINK1 occurs, which in turn ubiquitinates the phosphorylated outer mitochondrial membranous proteins, including Miro1 (which is phosphorylated at the Ser156 residue by PINK1 prior to its ubiquitination). The ubiquitinated proteins are then subjected to proteosomal degradation, thereby disconnecting the mitochondria from the microtubules and ceasing their transport [46]. Hsieh et al. demonstrated that LRRK2, a product from another PD-related gene, forms a complex with Miro on damaged mitochondria and helps in its removal, which leads to the initiation of the PINK1/PARKIN-mediated mitophagy. Mutated LRRK2 is unable to form the complex and hence delays Miro removal, which in turn retards the induction of PINK/PARKIN-mediated mitophagy [47]. In addition, the kinase activity of LRRK2 mediates mitochondrial fission and maintains a balanced autophagic flux by meddling with the cellular localization of lysosomes.Inhibition of this activity elongates the mitochondrial network and leads to poor degradation of the malfunctioning mitochondria [48, 49]. Studies conducted in *Drosophila* revealed that downregulation of dMiro leads to an increased survival rate of dopaminergic neurons in dPINK1-mutant phenotypes, whereas dMiro overexpression alone leads to DA neuronal death. Removal of Miro1 from depolarized and dysfunctional mitochondria is now being explored as a novel neuroprotective mechanism against PD, as reducing Miro1 levels has been shown to improve mitochondrial arrest and thereby mitophagy and prevent dopaminergic neuronal loss in iPSC-derived human neurons and *Drosophila* PD models without significant effects on the movement of healthy mitochondria [47, 50].

### Retrograde signaling

The axonal retrograde movement of mitochondria is driven by the cytoplasmic dynein motor protein. Unlike plus-end-directing kinesins, dynein-1 does not belong to any large family; rather, it is the only isoform that is universally conserved in all eukaryotic organisms, with flowering plants, red algae, and *Entamoeba* being exceptions, as they lack this protein [51]. In terms of its structural framework, it is more complex and bulkier than other motor proteins. It encompasses several polypeptide chains: two heavy chains, several intermediate chains, light intermediate chains and light chains. The heavy chain has two significant functional parts, an N-terminal tail that binds to intermediate chain, light intermediate chain and light chain, and a motor region that contains a series of hexaconjugated AAA^+^ (ATPases associated with various cellular activities) domains (AAA1 – AAA6) with each domain having its own unique function that is explicitly explained elsewhere [52], a microtubule-binding domain and a linker domain made of four α helical segments aiding in motility [53].

Mechanisms that link dynein to mitochondria are still under exploration, but cytoplasmic dynein typically requires a 11-subunit protein called dynactin for the regulation of long-distance motor movement over microtubules. ‘p150glued’, also known as DNCT1, is the largest subunit of dynactin, and it interacts directly with both cytoplasmic dynein and microtubules, thereby enhancing dynein processivity and its interactions with cargo proteins. Only the intermediate chain and heavy chain 1 of dynein as well as the three dynactin subunits Arp1 (α-centractin), p62, and p150Glued interact with mitochondria as well as other cargos [54]. A very recent finding indicates that the nanosized graphene oxide mediates the inhibition of attachment of the dynein-dynactin complex onto MTs and the subsequent inhibition of mitochondrial retrograde transport [55].The dynein-dynactin complex also associates with Bicaudal-D2 (BICD2) to increase its force production. A single dynein-dynactin-BICD2 (DDB) complex has the potential to successfully resist one kinesin module [56]. When both kinesin and dynein are stepping simultaneously on the MTs and are engaged in competing activity, the kinesin-1, -2 and -3 motors are observed to overpower the hindering forces exerted by the DDB complex via innate mechanochemical strategies and bias the overall cargo movement toward the plus-end [57]. The dynein light chain TCTEX1 has been reported to associate with the mitochondria via interactions with voltage-dependent anion selective channel 1 in the outer mitochondrial membrane [58]. Snapin is a dynein motor adaptor protein that binds to the dynein intermediate chain and enhances retrograde transport. The snapin-mediated retrograde transfer has recently been shown to reduce synaptic mitophagy stress and alleviate mitochondrial deficits, thereby improving synaptic conditions in AD mouse brain [59]. Functional loss of the *MIRO* gene impairs not only the anterograde movement but also the retrograde transport of mitochondria. Another protein regulating axonal transport of mitochondria is the Valosin-containing protein/p97 or VCP (an ATPase protein and an important elemental unit of the ubiquitin system). Upregulation of Miro elevates ATP production in VCP-mutant larvae of *Drosophila*. Downregulation of VCP results in enhanced mitochondrial retrograde transport and subsequently reduces its distribution in larval axons. Thus, VCP-relevant neurodegenerative diseases exhibit impaired mitochondrial transport [60]. Decreased VCP expression has been reported in late preclinical and early clinical stages of PD in untreated patients. This suggests that the decline in VCP mRNA expression is a potential biomarker for early clinical and preclinical PD pathology [61, 62]. MTX-2, miR-1 and TRAK-1 can form a distinct molecular adaptor unit and facilitate dynein-based mitochondrial retrograde transport [41]. Lissencephaly 1 (Lis1) or Neurodevelopment Protein 1 Like 1 (NDEL1) overexpression in rat dorsal root ganglion axons increases retrograde mitochondrial transport, while their knockdown or mutation results in inhibition of retrograde transport, indicating that Lis1 and NDEL1 are potential retrograde regulators [63]. Study by Kawano et al*.* further revealed that the deficiency of Lis1 triggers the accumulation of dynein and cargos in axonal terminals and NudC-mediated Lis1 stability is necessary for the regulatory maintenance of the dynamic MTs at the axon terminals, making Lis1 an indirect regulator of dynein [64]. Apart from being involved in axonal cargo transportation intraneuronally, dynein-dependent transport also plays a role in regulating the structural and positional organization of MT asters during mitosis [65].

**Fig. 2:**
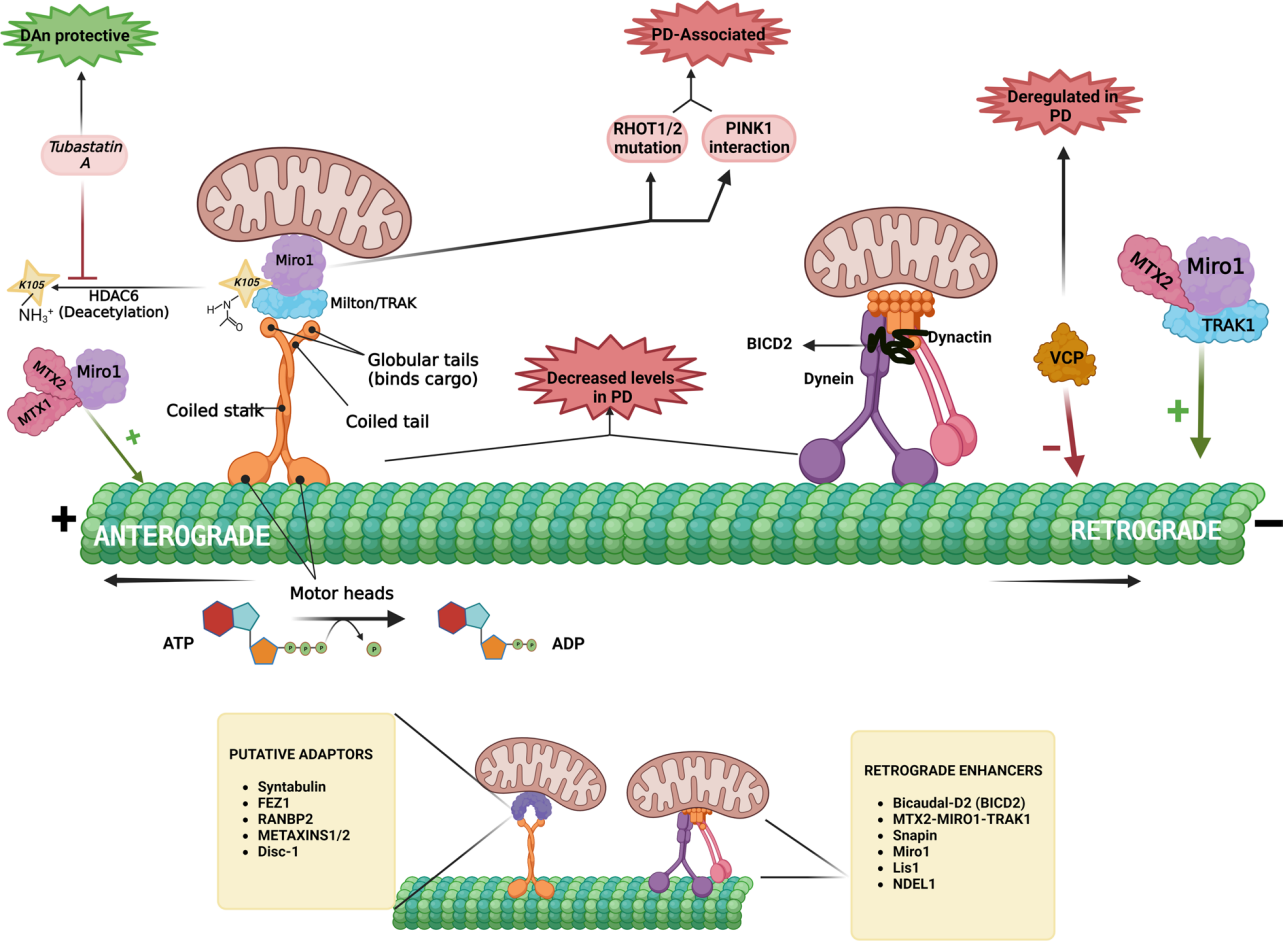
Microtubule (MT)-mediated axonal commute of mitochondria. The ‘plus’ end-directed anterograde transport of mitochondria is mediated by kinesin-1 motor protein, which is attached to the Miro1-Milton/TRAK adaptor complex. The rhythmic walking of its motor heads over MTs is driven by the hydrolysis of ATP molecules. Deacetylation of Miro1 by HDAC6 renders it functionally inactive, and HDAC6 inhibitors such as tubastatin A reverse it. Mutations in *RHOT1/2* (encoding Miro proteins) and deregulated Miro and PINK1 interactions are observed in PD pathogenesis. Various adaptors of the anterograde transport of mitochondria include syntabulin, FEZ1, RANBP2 (RAN-binding protein 2), METAXINS1/2 and Disc-1 (Disrupted in schizophrenia 1). Retrograde/ ‘minus’-end transport is facilitated by the dynein-dynactin-BICD2 complex. MTX1/MTX2/Miro1 promotes the retrograde movement of mitochondria, while MTX1/MTX2/TRAK2 promotes kinsein-1-mediated movement. The putative enhancers of mitochondrial retrograde signaling include BICD2, Snapin, Miro1, Lis1 and NDEL1. VCP downregulation observed in PD patients is a negative regulator of mitochondrial retrograde signaling. Both kinesins and dynein motor proteins responsible for axonal commute of various substances including vital organelles such as the mitochondria, seem to be dysregulated in neurodegenerative conditions such as PD

A large number of studies have linked the anterograde and retrograde axonal transport of mitochondria to neurodegenerative disease. Whether the impaired transport is the cause or the consequence of the disease is yet to be delineated, but the microtubule-mediated transport of essential organelles such as mitochondria and the regulation of the transport do play a role in neuronal health, and would result in several neuropathological conditions if hampered. The MT-mediated axonal trafficking is thoroughly depicted in Fig. 2.

## Neuronal MTs: tracing back to their genes

A great majority of the eukaryotic population expresses a unique set of genes for α- and β-tubulin, collectively called tubulin isotypes. Studies have identified 9 α-tubulin isotypes and 10 β-tubulin isotypes from the human genome. These isoforms can be discriminated on the basis of four main aspects, including chromosomal loci, amino acid sequences they encode, the 3’ untranslated region (UTR) nucleotide sequences, and their varying expression in different tissue types or development stages. The selective expression or suppression of these genes in different cell types contributes to the formation of different combinations of α- and β-tubulin heterodimers, which in turn interfere with the structural and functional behavior of MTs [66]. The majority of tubulin genes have been described to have different spatiotemporal expression patterns during different developmental stages in the mouse hippocampus [67]. Polymorphisms in α- and β-tubulin genes (*TUBA* and *TUBB*), such as neuron-specific beta3-tubulin (*TUBB3*) mutation, lead to congenital fibrosis of the extraocular muscles [68]. Other specific *TUBB3* mutations were found to result in impairment of α/β heterodimer formation, leading to lower stability of microtubules [69]. Alpha4a-tubulin (*TUBA4A*) mutations are strongly linked to familial ALS and frontotemporal dementia. The N-terminal mutations of the *TUBA4A* gene are considered to exert pathogenic defects via haploinsufficiency, while the C-terminal mutations disrupt MT homeostasis through a dominant-negative mechanism. A novel N-terminal TUBA4A frameshift mutation was recently discovered in an individual with a family history of Parkinsonism and exhibiting frontotemporal lobar degeneration [70]. TUBA1A is an intricate requirement during neuronal development for the assembly of microtubules required for neurite extension. Thus, mutations in this gene lead to severe cortical malformations along with serious neurological and physical phenotypes. In addition, a recent study has shown that a loss-of-function mutation in this gene results in adult-onset behavioral degeneration and disturbed microtubule assembly, leading to improper organelle trafficking and synaptic abnormalities [71]. A microarray-based gene expression profiling study in idiopathic PD patients revealed dysregulation of the following tubulin genes involved in synaptic plasticity: *TUBA1A*, *TUBB2A*, *TUBB2B*, *TUBB2*C, and *TUBB3* [72]. A recent study [73] showed that compromise to microtubule stability would lead to altered expression levels of TUBA and TUBB via modifications of the posttranscriptional regulation of tubulin mRNA. However, interestingly, the coregulation of other functional microtubule-interacting components, such as MAPs, motor proteins and  plus-tip-binding proteins, was not observed. An interesting observation in the study was that activation of the phosphatidylinositol-4,5-bisphosphate 3-kinase signaling stabilized tubulin mRNA by changing the microtubule dynamics. Impairment in microtubule stability has been correlated with several neurodegenerative conditions, such as PD, AD and ALS [30, 74–78].

## MAPs governing MT dynamics

The tubulin proteins possess (a) an N-terminal domain that regulates aspects of protein folding and contains a guanine nucleotide-binding region, (b) an intermediate residue and (c) a C-terminal domain that binds both MAPs and motor proteins [71]. MAPs are conventionally thought to be mere MT stabilizers, but emerging research suggests MAPs as the main, indispensable contributors to cytoskeletal organization, mitotic and meiotic spindle assembly, neuronal development and ciliary axoneme genesis [71]. Based on their innate functionality, the MAPs can be grouped into (a) motile motor proteins that regulate cargo movement across MTs, such as the kinesin and dynein family of proteins, which help in intracellular trafficking, as discussed in detail in the earlier sections, (b) MT depolymerizers and stabilizers that make and break the MT filaments, respectively, and (c) ‘plus’ and ‘minus’ end-binding proteins that govern MT dynamics by regulating MT growth and catastrophe. The intricate interactions between microtubules and MAPs can be deciphered by cryo-electron microscopy (cryo-EM), X-crystallography and nuclear magnetic resonance spectroscopy. Purification of tubulin proteins from brain extracts during the early 1970s led to the discovery of higher-molecular-weight proteins such as MAP1 and MAP2 and lower-molecular-weight tau. A decade later, MAP4 and MAP7 were copurified from HeLa cells. MAP6, previously known as STOP (stabletubule-only peptide), and MAP3, were identified as neuronal MAPs around the same time. MAP1C (dynein) was identified as a retrograde protein. Molecular cloning studies later revealed that MAP4 and MAP3 were the same protein and are now referred to as MAP4 [79]. MAP4 overexpression in hypoxic cells enhances cell viability by increasing ATP production. It stabilizes the MT architecture, modulates the interaction between dynein and VDAC and prevents mitochondrial permeabilization under hypoxic conditions [80]. Cloning of tau protein led to the discovery of six splice variants, and the splicing of tau mRNA has been widely explored as it is a major pathological hallmark in AD [81]. In late 1980s, MAP5 was isolated, but since MAP1 and MAP5 share significant similarities, they were renamed as MAP1A and MAP1B, respectively. Six mammalian homologs of echinoderm MAPs have been discovered by cDNA studies, including GLFND, MTR120, MAP8 (MAP1S), MAP9 and MAP10 that were discovered in the late 1990s and 2000s, and MAP11, a newly discovered MT-interacting protein whose silencing in SH-SY5Y cells causes decreased cell viability and proliferation. Mutations in MAP11 are associated with microcephaly in humans and zebrafish [82]. MAP6-knockout mice are used as models for studying cognitive deficits in schizophrenia. Accumulating studies have reported the involvement of MAP tau in various neurodegenerative diseases. For instance, hyperphosphorylation of tau triggers its delocalization from MTs, which is considered one of the hallmark features of AD [83]. Tau displacement from axonal MTs renders the MTs prone to MT-severing enzymes such as katanin. This increased susceptibility toward katanin-based MT severing might be a strong cause of several taupathies due to intense MT loss [84]. Recent research has shown the formation of tau islands on the surface of MTs, which prevent the interaction of katanin with MTs, offering protection against severing. However, these islands additionally block the MT–kinesin-1 interactions, which could potentially lead to impaired axonal transport of mitochondria given the role that kinesin-1 plays in the mitochondrial motility. Thus, the neuroprotective role of these tau islands remains controversial. MAP2 and MAP4 were also found to protect MTs from katanin-based severing in mammalian cells [85]. Another MAP known as doublecortin helps achieve the 13-protofilament configuration in developing neurons by actively binding to the ‘plus’ ends of the growing MTs. MAP7 was found to relieve the autoinhibition of kinesin-1, making it a positive regulator of motor proteins. MAP7 dysregulation has been observed in patients carrying the *LRRK2* G2019S mutant, the most common genetic cause of PD as seen in sporadic and familial PD patients. Increased MAP7 precipitates  dopaminergic neurodegeneration and neurite shortening in human-derived iPSCs, further suggesting its link with PD [86]. MAP7D1 regulates and maintains acetylated stable MTs, and MAP7D2 stabilizes MTs via direct binding. Both MAP7D1 and MAP7D2 facilitate MT stabilization by regulating cell motility and neurite outgrowth [87]. The gene *MAPT* codes for the protein Tau, a sophisticated MAP that has strong links with AD and PD [88, 89]. The tau–MT interaction regulates MT spatial assembly and dynamics, stabilizes MTs by conserving their long labile domains, and thereby governs the axonal transport [83]. A clinical trial is ongoing to explore the role of *MAPT* haploid H1b in autonomic dysfunction in PD (NCT05471713). Serum antibodies against the neuronal proteins Tau and tubulin have been found to be elevated in the sera of PD and AD patients compared to healthy subjects. Hence, serum antibodies for tubulin and tau could be regarded as potential biomarkers for identifying the specific neurodegenerative conditions at an early stage [90].

Another brain-dwelling MAP that has deep-rooted connections with PD and related synucleinopathies for decades is Tubulin Polymerization Promoting Protein (TPPP/p25). Although it does not possess a conventional, well-defined 3D structure, few distinct binding sites have been deduced. TPPP/p25 has a zinc finger domain, a GTP-binding segment and multiple phosphorylation sites [86]. It is a chief player in MT dynamics and stability maintenance and helps with the bundling of microtubules and concentration-dependent formation of defined microtubule ultrastructures. It binds to MTs and lowers the growth rate of ‘plus’ end, thus protecting the MTs from phases of depolymerization. TPPP/p25 increases MT acetylation via putative inhibition of HDAC6 [91]. It is generally overexpressed in oligodendroglial cells (OLGs) in the normal brain, but during synucleinopathies such as PD and multiple system atrophy, colocalization and abundant coexpression of α-syn and TPPP/p25 are observed in Lewy bodies and cytoplasmic glial cells. TPPP/p25-rich OLGs actively take up human α-syn preformed fibrils and form highly aggregated and insoluble pathological accumulations that are detrimental to MTs and the myelin sheath network [92]. This ectopically expressed protein in dopaminergic neurons during PD was determined to be a component of Lewy bodies purified from postmortem brains of PD patients [93]. TPPP/p25 knockout mice exhibit shorter lamellar microtubules. Although the neuronal morphology and axonal tracts remain highly intact, the myelin sheath is shorter and thinner. In addition, these KO mice also show great motor coordination deficits [94]. Ejlerskov et al. proposed that the aggregation and autophagosomal uptake of α-syn is a result of TPPP/p25 overexpression. The autophagosome and late endosome fuse to form an amphisome, which later travels retrogradely toward the lysosome and coalesces with it, forming an autolysosome, wherein α-syn is degraded. Retrograde transport requires HDAC6-mediated interactions and this enzyme is inhibited by TPPP/p25. The fusion of amphisomes and lysosomes to generate autolysosomes also requires HDAC6 deacetylase activity. Inhibition of HDAC6 activity by TPPP/p25 leads to the anterograde shift of the amphisome toward the cell body. Under the regulation of Rab27a, a portion of these amphisomes undergo exocytosis, releasing monomers and aggregates of α-syn into the extracellular space [95]. Both of the intrinsically disordered (not adopting a distinct native configuration in their isolated form) hallmark proteins α-syn and TPPP/p25 belong to the class of neomorphic moonlighting proteins, i.e., they have both physiological and pathological roles without changes at the genetic level; hence, targeting them for disease-modifying therapy would most likely turn out to be a challenge. An almost ideal solution to this challenge would be to target the interface of the TPPP/p25–α-syn pathological complex rather than the individual chameleon proteins (proteins displaying high conformational plasticity). The highly flexible CORE region of TPPP/p25 and the negatively charged C-terminus of α-syn interact with each other to form a pathological heterocomplex [96]. Recently, peptidomimetic foldamers, which are synthetic oligomers that mimic the conformational state of proteins and have the capability to bind the contact surface of protein complexes, have attracted attention in the field of drug research [97]. Proteolysis-targeting chimera (PROTAC) compounds can also be implemented for such interface targeting. Developed and patented PROTAC molecules exclusively targeting α-syn and β-amyloid currently do exist and can further target protein‒protein interactions [98].

## PTMs of tubulin – limelight on acetylation

The highly dynamic and complex eukaryotic cell is tightly regulated by a plethora of mechanisms, including epigenetic changes and PTMs. Epigenetic changes primarily include DNA methylation, chromatin remodeling orchestrated by histone modifications and RNA-driven regulation by noncoding microRNAs, siRNAs and long non-coding RNAs. For instance, the knockout of miR449 in healthy animals causes defective MT dynamics, and deregulated levels of miR449 are reported in PD models and PD patients [99]. While the epigenetic and epitranscriptomic regulation of tubulin protein has not been extensively explored thus far, the various PTMs it is subjected to have been widely studied.

**Fig. 3:**
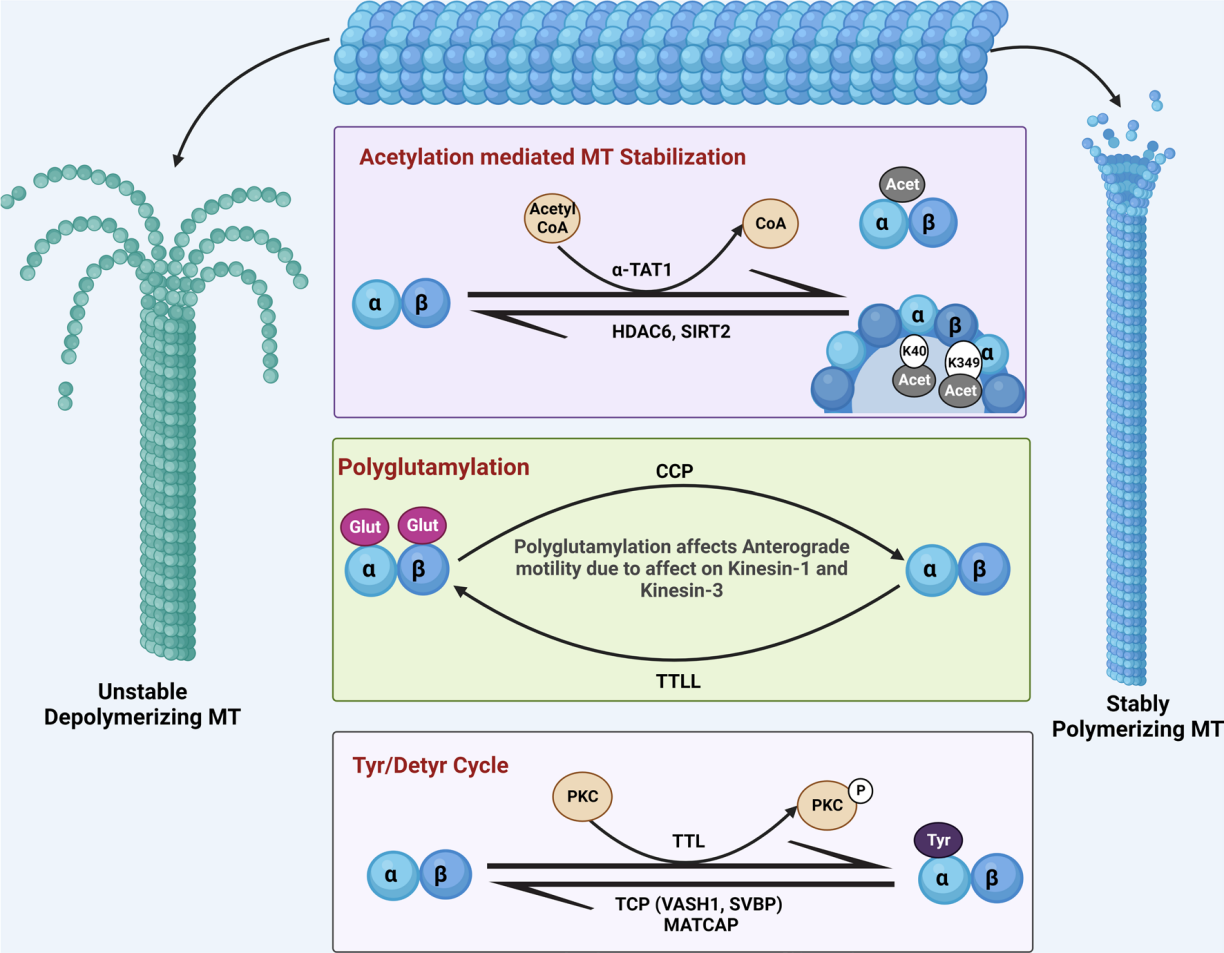
Post-translational modifications of tubulin. The luminal acetylation of K40 and K349 residues of the microtubules (MTs) via α–TAT1 imparts stability to them, and their subsequent deacetylation catalyzed by HDAC6 and SIRT2 makes them unstable by depolymerizing them. Polyglutamylation of tubulin, at the carboxy terminal tail of both tubulin monomers, is a negative regulator of anterograde axonal transport and is governed by the enzymes cytoplasmic carboxypeptidase deglutamylase (CCP) and tubulin tyrosine ligase like (TTLL). Stable tyrosinated MTs are regulated by the enyzyme tubulin tyrosine ligase (TTL) and deregulated to the detyrosinated version via tubulin carboxypeptidase (TCP), which is a complex of vasohibin-1 (VASH1), small vasohibin-binding protein (SVBP) and microtubule-associated tyrosine carboxypeptidase (MATCAP)

Regulatory PTMs are important for healthy functioning of physiological proteins. PTMs are generally characterized by proteolytic cleavage or addition of certain chemical moieties onto the surface of newly translated proteins to enhance or suppress their functionality. MTs are subjected to multiple PTMs. The structural integrity, stability and dynamics of MTs are heavily governed by PTMs of tubulin in its heterodimeric and protofilament configuration. Tyrosination, acetylation and polyglutamylation are some of the well-known modifications of tubulin that bring out the functional diversity of MTs (Fig. 3). Other less known yet significant PTMs include phosphorylation, polyamination, palmitoylation, S-nitrosylation, ubiquitylation, sumoylation, glycosylation, succination and methylation [100]. Detyrosination principally regulates the dynein-mediated retrograde motility, while acetylation accounts for kinesin-mediated anterograde microtubular transport [101].

### Detyrosination/tyrosination

In the early 1970s, a team of Argentinean scientists observed the incorporation of tyrosine into the α-tubulin C-terminus in a translation-independent manner in rat brain homogenates. Shortly after it, this reaction was found to be reversible by Hallak et al. [102, 103]. The detyrosination/tyrosination cycle of tubulin involves two sets of functionally opposite enzymes, tubulin carboxypeptidase (TCP) and microtubule-associated tyrosine carboxypeptidase (MATCAP), which are responsible for the removal of the C-terminal tyrosine moiety from α-tubulin, and tubulin tyrosine ligase (TTL) which facilitates re-addition. Chemical proteomics studies revealed that the major brain TCP is a complex formed by vasohibin-1 (VASH1) and small vasohibin-binding protein (SVBP) [104]. Recently, using cryo-EM, Li et al. deduced the structure of human VASH1-SVBP bound to MTs, and showed that VASH1-SVBP engages two adjacent α-tubulin molecules in neighboring protofilaments when binding to the surface of polymerized microtubules, thereby guiding the C-terminal tail of α-tubulin into the active site for tyrosine cleavage[105]. TTL re-adds tyrosine residues onto soluble tubulin dimers, and its activity is regulated through its phosphorylation by protein kinase C and its interaction with MAP1B, while VASH-SVBP and MATCAP act on MTs [106, 107]. *TTL*-heterozygous mice exhibit decreased tyrosinated MTs, reduced synaptic plasticity and dendritic spine density, and memory deficits. In addition, TTL was found to be downregulated in both sporadic and familial AD patients, and accumulations of detyrosinated tubulin have been observed in brain samples of AD patients [108]. Any compromise to tubulin tyrosination may lead to the manifestation of morphological abnormalities of the brain, such as microcephaly, cognitive defects as well as motor and speech impairment [109]**.** Newly polymerized MTs are tyrosinated, whereas stable MTs are highly detyrosinated. In the soma of neurons in particular, MTs are generally stable and detyrosinated, but in neuronal structures such as growth cones and dendritic spines, they remain highly dynamic and tyrosinated. Unlike other PTMs, tyrosination and detyrosination are highly specific only for α-tubulin, and the levels of tyrosinated MTs are relatively lower in neurodegenerative disorders [109]. Since only the newly polymerized MTs are tyrosinated, it remains unclear if MT tyrosination is indeeded altered in neurodegenerative conditions or if the observed lower MT tyrosination is due to the lower level of MT formation or polymerization. A recent study revealed a significant reduction of the tyrosinated state of MTs upon irreversible incorporation of *L*-dopa into tubulin, and subsequently, such prolonged alterations of the MTs affect its general functioning as well as mitochondrial trafficking, precipitating the development of *L*-dopa-induced dyskinesia [110]. Grignard et al. developed a mechanistic mathematical model of the microtubule detyrosination/tyrosination cycle by combining computational modeling and high-content image analyses to investigate the crucial kinetic parameters governing the tyrosination status in different cellular models. Two parametrizable models have been developed, one for neurons and the other for proliferative cells. Understanding the key kinetic parameters could eventually lead to ideas for increasing or decreasing the detyrosination/tyrosination status in such cellular models, which can be further translated to preclinical and clinical settings [111].

### Polyglutamylation

Polyglutamylation occurs at the C-terminal of tubulin at the outer surface of the microtubule. It is generally characterized by the addition of secondary peptide chains of variable numbers of glutamate to the primary peptide chains of tubulins, and is catalyzed by the family of polyglutamylases. The entire glutamylation reaction can be divided into two phases, initiation and elongation. Initiation starts in the primary protein chain, in which a covalent bond is formed between the γ-carboxyl group of the modified glutamate and the amine group of the added glutamate residue. During elongation, successive glutamate residues are added to the α-carboxy group of the antecedent glutamate [112]**.** The glutamylases targeting the tubulin proteins include tubulin tyrosine ligase-like (TTLL)1, 4, 5, 6, 7, 9, 11 and 13, while the deglutamylases include cytoplasmic carboxypeptidase deglutamylase (CCP)1, 4, 5 and 6. Neuronal nuclear protein induced by axotomy (Nna1) is the major neuronal deglutamylase, and its deficiency naturally causes the polyglutamylation of proteins and dysregulation of neuronal functioning [113]. *Nna1*-deficient mice show tubulin hyperglutamylation and ER dysfunction, which eventually lead to the demise of Purkinje neurons. A previous study showed that the Nna1 protein localized in mitochondria is involved in OXPHOS reactions and contributes to normal mitochondrial metabolism. Consistently, Purkinje cell degeneration mice display significantly reduced mitochondrial complex 1 activity [114]. With several studies indicating Purkinje cell death occurrence in PD [115, 116], can one assume the role of hyperpolyglutamylation of tubulin in the pathogenesis of neurodegenerative disorders such as PD? A study conducted in primary hippocampal neurons showed an elevation of tubulin polyglutamylation due to a deficit in the MT-severing enzyme spastin. Moreover, kinesin (KIF5 majorly)-mediated neuronal transport was impaired, and reducing the level of polyglutamylation reversed the affinity of kinesin toward the MT [117]. In contrast, another study showed a gain of function of kinesin-1 motor activity upon polyglutamylation of the carboxy terminal tails of both α- and β-tubulin [117]. Although not furnished in this particular study, the impairment of KIF5-mediated neuronal transport would most likely also affect mitochondrial trafficking, as these are the motor proteins involved in the axonal anterograde transport of mitochondria, as indicated by other studies. Impairment in KIF5-mediated axonal transport has also been directly linked with striatal dopaminergic neuronal loss [118, 119]. Embryonic primary neuron cultures lacking the *CCP1* gene showed immense tubulin hyperglutamylation, and the time expenditure of anterograde and retrograde mitochondrial movement was reduced by 50% without any compromise in the run length or speed of transport [120, 121]. Tubulin polyglutamylation also seems to negatively affect the lysosomal/endosomal particles in terms of the time spent in their axonal movement. Although mitochondria and lysosomal/endosomal particles are driven by kinesin-1 motors, the kinesin-3 motor protein-driven transport of BDNF is also affected in the presence of tubulin polyglutamylation, proving that hyperglutamylation does not exclusively regulate axonal transport of kinesin-1-driven cargos [121].

### Acetylation

Acetylation is one of the most studied PTMs of tubulin or any other protein in general. In the past, acetylation was thought to be restricted to the histone proteins in the nucleus, but more than three decades of research has unraveled the vital role this PTM plays in the regulation of nearly one thousand non-histone proteins. Acetylation of MTs typically refers to the transfer of acetyl groups onto the ε–amino group of lysine 40 (K40) in α-tubulin from an acetyl coenzyme A moiety. K40 acetylation of α-tubulin is so far the only known PTM occurring on the luminal side of MT and hence is referred to as the ‘hidden’ PTM [122]. This K40 acetylation has been evidenced as an evolutionarily conserved PTM in a wide range of organisms ranging from protists to the human beings. Shortly after its discovery in the flagella of *Chlamydomonas reinhardtii*, researchers wondered whether α-tubulin acetylation is the cause or a consequence of MT stability. Novel molecular dynamic methods and atomic-resolution cryo-EM maps have precisely elucidated restriction of the range of motion of the αK40 loop upon acetylation. Such an entrapment of the loop in a much rigid, immovable structural ensemble prevents it from establishing lateral contacts and thereby imparts structural stability. Such data imply a causative relationship between MT acetylation and stability [123]. Proteomic profiling of tissues from varying sources, such as humans, rodents and flies, has unraveled multiple (at least 12) evolutionarily conserved sites on α-tubulin that are prone to acetylation [100, 124–126]. A single site spotted unanimously in all these organisms is the K394 domain in α-tubulin. Unlike the K40 domain, which sits on the luminal surface of MTs, K394 is located on the interface of the α/β-heterodimer, and its acetylation seems to modulate MT stability during neuronal synaptic bouton morphogenesis. In an acetylation-blocked K394R mutant fly model, motoneuron morphogenesis was found to be disorganized along with compromised MT stability, further emphasizing the correlation between MT stability and α-tubulin acetylation [127]. Apart from imparting stability, acetylation also enhances the survival of MTs, contributing to their longer lifespan and increasing resistance to strain, flexibility and the ability to be easily repaired if damaged [128]. Additional studies have highlighted that acetylated MTs promote and improve motor-based cargo trafficking [13, 129]. Acetylation also increases the MT lifespan by increasing its endurance to mechanical stress [128]. MTs that are acetylated tend to bundle predominantly, and such MT bundles associate with abundant motor proteins, including kinesin-1, due to the inherent increase in the proportion of binding sites [130]. The mechanical movement of kinesins along the MT tracks has the ability to inflict damage to the MT lattice; for instance, a mutated kinesin-1 motor (KIF5C) generates high amounts of damage sites on the MT lattice when compared to the wild-type motor, but the acetylated MTs would remain immune to such mechanical turmoil [101]. Verhey and colleagues furnished strong evidence that acetylated MTs provide incredible support for kinesin-driven anterograde transport of JIP-1 to the tips of neurites [13]. Hyperacetylation of MTs is an early response observed when cells cope with internal stress signals. It usually results in enhanced kinesin-1-mediated movement of JNK and subsequent translocation of total Drp-1 to the mitochondrial preconstriction sites, promoting its fission process [131]. In contrast, findings from a recent study imply that psychological stress induces a reduction of tubulin acetylation level in prefrontal cortex of mice and the treatment of CB1 receptor agonist WIN55,212–2 increases  it. Other studies have reported neuroprotective effects of WIN55,212–2 on striatal neurons of a 6-OHDA PD model by altering the ERK1/2 phosphorylation status [132] and in an 1-methyl-4-phenyl-1,2,3,6-tetrahydropyridine (MPTP)-induced PD model by suppressing microglial activation [133]. Apart from this, WIN55,212–2 was found to increase dopamine and 3,4-dihydroxyphenylacetic acid levels in striatal neurons [134]. Tubulin acetylation was also found to enhance the penetrative capacity of cells undergoing radial intercalation, a physiological process in which cells move apically and join an epithelium by inserting throughout the thickness of a multilayered tissue [131]. Complete loss or reduction of α-tubulin acetylation in neurons has been highly correlated with the manifestation of numerous neuropathological conditions, such as AD, PD, HD, Charcot-Marie-Tooth disease, and familial dysautonomia [135–138]. Hence, acetylation plays a vital role in anterograde motility-related pathological development in neurodegenerative diseases such as AD and PD. Strategies targeting this core process need to be explored in detail in the future.

#### Enzymes governing acetyl-mediated tubulin modifications

##### α-Tubulin acetyl transferase (α-TAT1)

The enzymatic regulation of α-tubulin acetylation is achieved by the major acetylator α-TAT1 and two deacetylators, namely, HDAC6 and SIRT2. To date, at least 22 lysine acetyltransferases (KATs) have been identified in humans and they are grouped into three major families: the MYST family, the general control of amino acid synthesis 5 (GCN5)-related *N*-acetyltransferases (GNAT) family, and the p300/CBP family. The prominently distinguished α-tubulin acetylator α-TAT1 belongs to the GNAT family of KATs, and in addition to this enzyme, MEC17 (*Caenorhabditis elegans* protein mechanosensory abnormality 17), ARD1–NAT1 (arrest defective 1–aminoterminal, α-amino, acetyltransferase 1), the ELP complex (elongator protein complex) and GCN5 are also regarded as indirect candidate catalysts of α-tubulin K40 acetylation [139, 140]. Several KATs are reported to be deregulated in PD and are implicated in pathological involvement at some level [101]. A lack of detectable levels of K40 α-tubulin acetylation was observed in MEC17/α-TAT1-deficient mice. The deacetylation state was not increased by the other enzymes, such as ARD1-NAT1, ELP3 or GCN5, proving that they are not direct acetylators of α-tubulin and hence cannot compensate for the loss of α-TAT1 [140]. Optimum levels of MEC-17 and ATAT-2 are needed for the temporal control of synaptic branching. Overexpression or loss of these enzymes causes a delay in synaptic branching and impaired synaptic formation in mechanosensory neurons of *Caenorhabditis elegans* [141]. Neuronal cells with defective elongators show a drastic reduction of acetylated α-tubulin. The ELP-3 catalytic domain of the elongator complex promotes acetylation and counteracts HDAC6-mediated deacetylation *in vitro*. α-Tubulin is a target of the elongator complex [139]. GCN5/KAT2A, although regarded as a histone acetyltransferase enzyme, has catalytic activity over MT as well. It modulates axonal outgrowth by regulating the proportion of acetylated tubulin [142]. While histone acetyltransferase enzymes have been largely explored, there is a lack of structural and mechanistic data regarding α-TAT1. In comparison with the well elucidated KATs, α-TAT1 possesses a highly conserved co-substrate-binding domain that is unique in dual aspects, one being the active site and the other being the putative α-tubulin-binding site. A conserved glutamine residue was deduced as the propelling force behind the displayed catalytic activity of the enzyme [143]. Friedmann et al. demonstrated that highly conserved aspartic acid and cysteine residues, D157 and C120, located on the active site of the enzyme, perform catalytic activity through the formation of a ternary complex. In comparison with GCN5 histone and Naa50p *N*-amino acetyltransferases, α-TAT1 possesses a relatively wider substrate-binding groove of 20 Å [144]. Whole protein docking was performed by Nung-Yu Hsu et al. to elucidate the different binding zones in the α-TAT1 crystal structure, and pharmacophore anchor models developed using SiMMap revealed three binding subpockets, namely, the S1 acetyl site, the S2 adenine site and the S3 diphosphate site. Validation of their model performed via BLAST across the α-TAT1 sequences of 14 species showed that the Q58, D157 and R158 residues were conserved at the S1 site. Mutations of D157E, R158A and Q58A induced a reduction/loss of the K40 acetyltransferase activity of α-TAT1 [144–146]. Another highly conserved residue in the S2 site was identified as R132, and its mutation (R132A) decreased the enzymatic activity of α-TAT1 to 50% of wild-type activity [145]. R132, H133 and G134 of the S3 diphosphate site are also highly conserved and are capable of forming hydrogen-bond interactions [147]. Depletion of one of the cip/kip family members, p27^Kip1^, is correlated with decreased α-tubulin acetylation. p27^Kip1^ promotes the stabilization and regulation of α-TAT1 via binding to its highly conserved C-terminal domain, and its loss results in reduced levels of α-TAT1, leading to a subsequent decrease of α-tubulin acetylation and manifestation of axonal transport defects [148]. α-TAT1 is hypothesized to act as a clock representing the life span of MTs [122], and its deficiency causes an increase in the frequency of its mechanical breakage [128]. By scanning the MT bidirectionally, Szyk et al. found that α-TAT1 acetylates the tubulin moieties without having any bias toward a particular end [122]. Interestingly, α-TAT1 was found to have a self-acetylating property that regulates its function at α-tubulin. It has been established that α-TAT1 has specific affinity toward acetylating the α-tubulin of the MT and lacks acetylating activity toward histone protein substrates [149]. The enzyme is considered to reach the acetylation site present on the luminal side, either by entering through the exposed ends of the MTs or by sneaking in through the crevices of structural irregularities in the MT framework [150]. The precise molecular mechanism of luminal entry is an area under active investigation and requires further exploration. A recent study revealed that the active transport of α-TAT1-rich vesicles in axons is the predominant driving force for axonal MT acetylation. It was also demonstrated that precise bidirectional vesicular transport requires proper α-tubulin acetylation, mediated via α-TAT1, and functional loss of this enzyme leads to impaired axonal transport in neurons [151]. Increased α-TAT1-mediated MT acetylation was found to be the cause of breast cancer cell metastasis [152], while knockout of α-TAT1 in colon cancer cells inhibits their proliferation and invasive migration potential [153]. In the case of neurodegenerative conditions such as AD and PD, an increase in MT acetylation was found to benefit MT dynamics and enhance the binding of MAPs such as α-syn and tau with tubulin tracks [78, 154]. MTs are promising therapeutic targets for neurodegenerative diseases. While a small number of α-TAT1 inhibitors exist, there are no moieties that can activate α-TAT1 hitherto [155]. Hence, an in-depth comprehensive exploration regarding the molecular basis of α-TAT1-mediated MT acetylation could potentially open the doorway for designing small-molecule modulators that enhance or reduce the acetylation phenomena of MT tracks.

##### Erasers of microtubule acetylation (HDACs)

In total, there are 18 human HDAC enzymes, which, upon their equivalent homology with the yeast HDACs, are classified into four classes, classes I–IV. Class I Rpd3-like proteins include HDAC1, HDAC2, HDAC3, and HDAC8 with highly conserved deacetylase domains possessing strong affinity toward histone proteins in the nucleus, and their activation is monitored by inositol phosphates [156]. Their activity is not limited to histones but further extends toward non-histone proteins, including AMP-activated protein kinase and cohesion subunit SMC3. Class II comprises Hda-1-like proteins including HDAC4, HDAC5, HDAC7, HDAC9, HDAC6, and HDAC10, which are further grouped into isozymes IIa (HDAC4, HDAC5, HDAC7, and HDAC9) and IIb (HDAC6 and HDAC10). Both class I and II HDACs that are homologous to yeast Rpd3 and Hda1, respectively, have Zn^2+^-dependent domains regulating their catalytic activity. The N-terminal domains of the class IIa HDACs harbor a binding site for MEF2, a DNA-binding transcription factor, and have a conserved Ser residue, which makes the enzymes frequently subjected to phosphorylation via different kinases, such as microtubule affinity regulating kinase and calcium/calmodulin-dependent protein kinase. Phosphorylation promotes the nuclear localization of HDACs [157, 158]. The enzymes of class IIa have low enzymatic activity due to the replacement of the catalytically active and conserved tyrosine residue with the much less active histidine moiety [159]. These class IIa HDACs are found to engage in the formation of a large complex with the SMRT/N-CoR-HDAC3 complex to enhance transcriptional repression in the nucleus [160]. A recent study reported the accumulation of HDAC5 in the nucleus of 6-OHDA-treated dopaminergic cultures, which further induces neurite shortening and microglial activation. MC1568 selectively inhibits HDAC5 and prevents its detrimental effects in 6-OHDA-lesioned rats [161]. Class IIa-specific HDAC inhibitors promote neurite outgrowth, and the inhibition of HDAC4/5 by LMK235 exerts neuroprotective effects against DA degeneration in MPP^+^-treated SH-SY5Y cells as well as cultured DA neurons [162]. Class IIb enzymes (HDAC6 and HDAC10) have an unusually long extending C-terminus called the tail domain. HDAC6 and HDAC10, although belonging to the very same class, are different in their structures. HDAC6 possesses a zinc finger ubiquitin-binding domain at the C-terminal end and two deacetylase catalytic domains CD-1 and CD-2, while HDAC10 has only one deacetylase domain and a leucine-rich repeat domain at its C-end, and both of the enzymes are present in the cytoplasm. Additionally, the CD-1 domain of HDAC6 exhibits E3 ubiquitin ligase activity [159]. Apart from having a role in the deacetylation of several proteins including α-tubulin, cortactin, chaperones, and IFNαR, HDAC6 is also involved in the regulation of autophagy and hepatic metabolism [163, 164]. The disordered N-terminus of HDAC6 functions as the MT-binding domain and promotes chemotactic cell motility [165]. In vitro and in vivo studies have shown the involvement of HDAC6 in the deacetylation of α- and β-tubulin. Upregulation of HDAC6 in mammalian cells induces tubulin hypoacetylation, while its inhibition by pharmacological or genetic interactions causes microtubule hyperacetylation [166–169].

Biochemical analyses of tubulin deacetylation via HDAC6 revealed  its strong preference  for tubulin dimers to assemble MTs. Although HDAC6 is believed to deacetylate the K40 residue of α-tubulin, quantitative mass spectrometry studies revealed K60, K370, and K394 on α-tubulin as well as K58, K103, and K154 on β-tubulin as newer putative sites of HDAC6-mediated deacetylation [141]. A previous study showed that increased MT acetylation via HDAC6 inhibition leads to the recruitment of molecular motors kinesin-1 and cytoplasmic dynein to MTs, thereby increasing the MT-dependent corticostriatal transport of BDNF [137]. A septin-dependent manner of neurite growth extension is governed by HDAC6. SEPT7 offers a mechanical scaffold for HDAC6 to attach to acetylated α-tubulin and mediate its deacetylation, and thereby negatively regulates MT stability [170]. HDAC6 inhibition has been demonstrated to rescue decreased acetylation and impaired neuronal trafficking in multiple experimental models. Highly prominent intercellular Hedgehog signaling (Hh) increases the MT-associated DYRK1B kinase levels, which subsequently leads to the decrease of HDAC6 level and thereby modulates MT acetylation. DYRK1B-mediated Hh signaling activation also increases the intracellular transport of mitochondria, implying the beneficial role of acetylated MTs in organelle transport [171]. All these findings signify the critical role of tubulin acetylation in the pathological framework of diseases [26, 149, 167, 172]. The polyamine deacetylase, HDAC10, catalyzes the hydrolysis of N8 acetylspermidine to acetate. In alignment with the class IIa HDACs, HDAC10 is also assumed to form a complex with HDAC3, SMRT, and N-CoR to regulate gene transcription [173–175].

Sirtuin proteins are grouped within the class 3 HDACs and include SIRT1–7. They are NAD^+^-dependent enzymes with different subcellular localizations and are widely distributed over the cytoplasm, nucleus and mitochondria [176]. SIRT1 and 2 are present in all three places, with SIRT2 having particular rich expression in the plasma membrane and cytoskeleton-associated organelles. The localization of NAD(P)H:quinone oxidoreductase 1 (NQO1) near MTs leads to the hypothesis that the NQO1-catalyzed oxidation of NADH to NAD^+^ may drive the deacetylase activity of SIRT2, according to the results of a novel study [177]. While HDAC6 is known to regulate the majority of K40 acetylation, SIRT2 modulates K40 acetylation in the perinuclear region. SIRT2 contains a CRM-1 (chromosomal maintenance-1)-dependent nuclear export signal (NES) and a leucine-rich NES [178, 179]. SIRT2 shuttles back and forth from the nucleus to the cytoplasm since its substrates are widely distributed throughout the cell. These substrates include p53, FOXO1, FOXO3a, histone H4, histone H3, and p300 and have implications in various diseases [180]. Several studies based on environmental or genetic PD models demonstrated that deletion of SIRT2 protects against dopaminergic neuronal loss [181–183]. Recent research proves that the translocation of SIRT2 into the nucleus is controlled by Cdk5-mediated phosphorylation at residues Ser331 and Ser335 in its structural conformation. Inhibition of this phosphorylation by myristic acid-conjugated short peptide Myr-SIRT2_328–339_ seemed to display neuroprotection by rescuing MPTP-treated primary neuronal cells [184]. High levels of SIRT2 and low level of αK40 acetylation lead to improper organization of the MT in a 5 × FAD mouse model of AD [185]. SIRT3, SIRT4 and SIRT5 are densely populated in the mitochondria, and SIRT3 can also be shuttled between the mitochondria and nucleus. SIRT6 is mainly localized in the nucleus and structurally lacks the highly conserved NAD^+^-binding loop, making it a slowly catalyzing enzyme in comparison with the other active SIRTs. SIRT7 is distributed in both the nucleus and the cytoplasm [186] and is involved in mitochondrial regulation [187]. The final class of HDACs contains only one enzyme, HDAC11, and recent studies have highlighted its importance in the phenomenon of defatty-acylation of proteins [188]. While HDAC11 is also a Zn^2+^-dependent enzyme, it has been classified under a different class, as it does not contain amino acid sequences similar to the other classes.

## MT acetylation status in PD

To date, there is a large amount of *in vitro* and *in vivo* evidence pinpointing the defective regulation of tubulin acetylation in multiple neurodegenerative disorders, including AD and PD [78, 154, 189–191]. However, research on the mechanisms underlying the impaired MT functioning in PD is still in its infancy. Any malfunctioning of the MT framework may play a potential role in the development of PD, as MTs are crucial for healthy neuronal performance. MTs exert various functions, such as in organelle and protein trafficking, cellular differentiation, cargo transport, and motility [190, 191]. For a long time now, oxidative stress has been a major and constant culprit in PD pathogenesis, and this has been supported by several postmortem studies in PD brains showing increased levels of oxidative proteins, lipids and nucleic acids [192–196], as well as in studies of genetic or toxin-induced PD models[197–201]. In non-neuronal cells, researchers found that oxidative stress affects MT dynamics by hindering tubulin polymerization and hence affects its stability [202–204]. In a 6-OHDA-induced PD model, the levels of polymerized tubulin were found to be deregulated, and MT-dependent transcription factors were observed to be impaired [205, 206]. 6-OHDA-induced sublethal oxidative stress leads to a substantial decrease of the MT growth rate, leading to neurite shortening, which is one of the important phenotypes in the early stages of PD [207]. Oxidative stress in the form of mitochondrial ROS potentially enhances the levels of free tubulin, which in turn interacts with α-syn and promotes the formation of oligomeric aggregates. A very recent study revealed a significant decrease in acetylation of tubulin and MAP tau in post-mortem brains of PD patients (Braak stage IV-VI) exclusively in the SNpc, strengthening the relationship between MT acetylation and PD pathology [77]. Deficiency of the PD-associated protein Parkin was found to negatively regulate MT stability in both nerve growth factor-differentiated PC12 cells and PD patient-derived TH^+^  neurons. Defective MT functioning due to *PARK2* (which codes for Parkin) silencing also induces altered mitochondrial transport. Parkin is reported to influence MT stability by binding to the tubulin moiety via three interacting domains. While the precise mechanism of the MT stability is not well established, it is speculated that the binding to MTs provides a terrain for Parkin to perform its E3 ligase activity on misfolded proteins that are transported via MTs [25]. Parkin additionally decreases MT depolymerization via decease of MAP kinase activity [208]. The *LRRK2* gene, which is highly correlated with PD, is widely present with pathogenic Roc-COR domain mutations (R1441C and Y1699C). These mutant forms can bind to deacetylated MTs with great affinity and halt axonal transport in primary neurons and in a *Drosophila* model by interfering with the tethering of molecular motors to MTs, causing locomotor deficits [209]. Cryo-electron tomography has shown that the conformation of the kinase domain of catalytic half of LRRK2 regulates its interactions with MTs, and this in turn influences the binding of molecular motors such as kinesin 1 and cytoplasmic dynein 1 onto the MTs in vitro. Treatment with type II LRRK2 inhibitors that stabilize an open conformation of the LRRK2 catalytic domain improves the motility of kinesin and dynein in vitro [210]. A long-believed notion is that synaptic-axonal dysfunction and terminal degeneration precede the nigral dopaminergic loss, and LRRK2 biology definitely plays a role in it [211]. Inhibition of MT deacetylators such as HDAC6 and SIRT2 or overexpression of α-TAT significantly reduces the interaction of mutated LRRK2 filament with MTs [209]. LRRK2 mutants reduce neurite outgrowth and cause an accumulation of hyperphosphorylated tau. The specific interactions of LRRK2 with the β-tubulin isoforms TUBB, TUBB4 and TUBB6 regulate the luminal acetylation pattern of α-tubulin. Alterations of MT stability mediated by LRRK2 mutant–tubulin interaction may potentially be one of the pathogenic pathways promoting PD [212].

The α-syn-induced neurotoxicity in PD models can be reversed by enhancing MT acetylation via SIRT2 inhibition [181]. In 2,5-hexanedione-treated mice, the number of dynamic MTs was reduced in the soma of nigral dopaminergic neurons. While the distribution of acetylated MTs was affected by the treatment, the level of the acetylated MTs was unchanged [78]. However, another study showed decreased levels of acetylated α-tubulin and impaired mitochondrial trafficking [213]. Acetylation is also the most predominant PTM in astrocytes. A most recent study has demonstrated the importance of acetylated tubulin in astrocytes in the process of wound healing and tissue repair. Astrocytes migrate to the region of the wound and aid in its healing. Knockout of α-TAT in mouse models resulted in irregular migratory patterns of astrocytes and therefore compromised wound closure [214]. α-Tubulin acetylation induced by pharmacological intervention leads to a reduction in astrocyte reactivity and promotes the survival of SNpc dopaminergic neurons [215]. These results further highlight the link between PD and MT malfunctioning. While these results suggest the lack of acetylation as one of the contributing factors for PD, there are PD-related toxin studies that prove otherwise. Hyperacetylation of MTs due to an age-dependent decrease in Sirt2 activity has been shown to cause impairment of MT-based transport [216]. Similarly, the PD-inducing toxin MPTP was also found to hyperacetylate neuronal MTs, leading to impaired axonal transport in cells and mouse models [7, 217]. MPTP-injected mice show a drastic increase in acetylated tubulin level in the cell bodies of dopaminergic neurons in the SNpc [7]. Ex vivo cybrid cell studies (fusion of isolated mitochondria from PD patient platelets into mitochondria-deficient NT2 neuronal cells) showed a compromised MT network and a highly increased free/polymerized tubulin ratio [77]. Functional loss of the X-linked methyl-CpG-binding protein 2 (*MECP2*) gene causes Rett syndrome, a rare neurodevelopmental disorder. Malfunctioning of the *MECP2* gene is associated with decreased level of α-tubulin acetylation leading to impaired MT dynamics, which can be reversed by HDAC6 inhibitors [218]. Loss of *MECP2* causes a disruption of the nigrostriatal pathway in SNpc dopaminergic neurons and contributes to the parkinsonism-like symptoms seen in Rett syndrome. Furthermore, MECP2 and HDAC6 were found to function as epigenetic factors regulating α-tubulin acetylation in cardiac fibroblasts [219, 220]. Therefore, molecules modulating MECP2 by targeting HDAC6 may be a promising intervention in neurological conditions where MT acetylation dyshomeostasis is observed.

Several studies converge on the common idea that increasing the tubulin acetylation could be the ultimate remedy for major neurodegenerative conditions, including PD and AD. Although there has been mounting evidence for its role in neuropathological perturbations, whether it serves as a protective or destructive mechanism remains unclear. While most studies have reported the role of the lack of α-tubulin acetylation in impairment of axonal transport and neuronal degeneration in PD (as seen above), there are also contradictory findings [217, 221, 222]. These different results may be due to the variations of neurodegenerative models, the time point of analyses, the disease stage at study, and the course of disease progression. The observed increase in α-tubulin acetylation in the diseased models could also be interpreted as one of the protective mechanisms engaged in enhancing MT stability to facilitate precise axonal transport.

## Therapeutic strategies for enhancing tubulin acetylation

### HDAC6 inhibitors

It has been repeatedly established that a low level of tubulin acetylation may lead to neurodegenerative conditions. Attempts to tackle this situation can be made by utilizing tubulin acetylation enhancers. This can be achieved by blocking those enzymes responsible for their deacetylation, such as HDAC6 and SIRT2.

Kawaguchi et al. observed colocalization of α-syn and HDAC6 in Lewy bodies. Additional cell culture and in vivo studies revealing the coupling of HDAC6 with the K-63-linked polyubiquitinated mutant DJ-1 (protein associated with early familial PD) further strengthen the role of HDAC6-mediated deacetylation in PD [223]. An additional transcriptome study of clinical PD patient samples revealed a 1.6-fold increase in HDAC6 level [224]. A multidimensional screening of 7392 small molecules discovered the first selective HDAC inhibitor, tubacin. Tubacin solely targets HDAC6 and exerts its effects on tubulin acetylation without any effect on histone proteins [225]. Godena et al. found that PD-associated *LRRK2* mutations inhibit axonal transport and cause locomotor deficits; these changes were found to be significantly reversed by HDAC6 inhibitors such as trichostatin A, tubastatin A (TBA) and a SIRT2 inhibitor, AGK2 [209]. TBA-mediated selective inhibition of HDAC6 has proven to be neuroprotective for dopaminergic neurons in the SNpc, and can remarkably prevent the formation of the toxic Ser129-phosphorylated α-syn. In addition, a marked enhancement in chaperone-mediated autophagy activation and reduction in neuroinflammation (by decreasing astrocyte reactivity) have been observed [215]. Additionally, TBA attenuates the formation of the NLRP3 inflammasome and thereby alleviates dopaminergic neurodegeneration and glial proliferation by modulating the acetylation of peroxiredoxin 2 [226]. Rutin, a natural flavonoid, has been reported to be protective against PD [227–229]. A new study demonstrated rutin-mediated inhibition of HDAC6 without any toxicity and thereby a significant increase in α-tubulin acetylation levels in motor neuron-like NSC34 cells, uncovering a new mechanistic approach [230]. Venlafaxine, an antidepressant, has HDAC6-inhibiting properties, and inhibition of striatal HDAC6 increases the autophagic clearance of α-syn, restores the lost striatal dopamine levels and preserves dopaminergic neurons in a rotenone-induced rat PD model. The anti-depressant activity it possesses also comes in importance, as depression is one of the most debilitating nonmotor symptoms observed in PD [231]. T-3796106 and T-3793168 are two highly potent and low-toxicity novel HDAC6 inhibitors found to significantly increase MT acetylation even at nanomolar concentrations. They seem to rescue the impaired axonal mitochondrial transport in the primary neuronal culture model of Charcot-Marie-Tooth Type 2F. T-3793168 drastically enhanced the axonal anterograde and retrograde flux of mitochondria in the pathological model and showed no effects in the wild-type cells. Additionally, both drugs increase the human whole-blood level of α-tubulin acetylation [12]. The pan HDAC inhibitor vorinostat (SAHA) rescues α-syn-induced dopaminergic degeneration in a PD model, but the exact mechanism of action is unclear. Since it is not exclusively HDAC6-selective, plausible mechanisms would be increased acetylation of histone proteins as well as non-histone proteins such as tubulin [232–234]. SAHA has proceeded to a phase 1 clinical trial for AD (NCT03056495), and a recruiting study in PD patients has been designed at a very small and minimal scale (NCT03977740). Increase of acetylated tubulin levels by yet another small-molecule HDAC6 inhibitor, ACY-1215, leads to a parallel significant increase of speed and track length of mitochondrial transport [171]. The novel compound *N*-[(1R,2R)-2-{3-[5-(difluoromethyl)-1,3,4-oxadiazol-2-yl]-5-oxo-5H,6H,7H-pyrrolo[3,4-b]pyridin-6-yl}cyclohexyl]-2,2,3,3,3-pentafluoropropanamide, T-518 in short, is a potent and highly selective HDAC6 inhibitor with clinically favorable pharmacodynamics that induces a significant increase in tubulin acetylation in the hippocampus of P301S tau transgenic mice. A two-week oral treatment with T-518 was shown to restore axonal transport and reduce aggregated insoluble tau, making it a promising candidate for posterity [235]. Treatment with NCT-14b in HeLa cells exclusively elevates the level of α-tubulin acetylation, proving that it is a highly selective HDAC6 inhibitor [236]. Docking simulation studies revealed the putative antagonistic interactions of compound ‘1–8-6’ (benzopyrazole derivative) with the active catalytic sites of HDAC6, leading to enhanced expression of acetylated α-tubulin, making it a promising candidate for future preclinical trials [237]. In human AML cells, the 2-benzyl-amino-naphthoquinonecompound triggers the hyperacetylation of tubulin more quickly when compared with TBA. Vitamin K3 (2-methyl-naphthoquinone) was also found to induce the acetylation of α-tubulin at a lower micromolar concentration but it is selective toward both HDAC6 and HDAC8 [238]. Recent research conducted by Hye Yun Chae et al. established the α-tubulin-acetylating property of ‘compound 6e’ via HDAC6 inhibition in addition to Hsp90 inhibition [239]. Molecules with a diphenyl-azetidin-2-one scaffold seem to possess dual hHDAC6/hHDAC8 inhibition properties and hence increase α-tubulin acetylation [240].

### SIRT2 inhibitors

SIRT2 inhibitors are the other class of drugs that could be implemented to enhance α-tubulin acetylation and hence may be neuroprotective agents in neuronal conditions where MT acetylation is severely compromised. Altered NAD + metabolism observed in sporadic PD patient-derived cells results in the activation of SIRT2 which subsequently reduces the level of acetylated tubulin and causes an imbalance in the trafficking and clearance of misfolded proteins, impairing MT assembly and the neuronal autophagic flux. Knockout of SIRT2 in these cells reverses the conditions and promotes neuronal survival [241]. The compound ICL-SIRT078 rescues the lactacystin-induced N27 cell lines from neuronal death by inducing tubulin acetylation and FOXO3a accumulation [183]. Inhibitor of DNA binding 2 (Id2) competitively blocks the interaction between SIRT2 and α-tubulin by binding to the α-tubulin intermediate domain, thereby increasing the levels of αK40 acetylation. Upregulation of Id2 may be a novel strategy to enhance α-tubulin acetylation [185]. Additionally, Id2 promotes axonal growth in neurons by inducing the upregulation of Neurogenin2, making it a potential therapeutic target in neurological conditions with tubulin or axonal deficits [242]. AGK2 (a propenamide derivative) treatment induces a dose-dependent increase in MT stability via enhanced α-tubulin acetylation in bovine brains. AGK2 is a quinoline derivative and a potent inhibitor of the SIRT2 deacetylase enzyme. AGK2 and AK1 rescue the α-syn-mediated toxicity to dorsomedial dopamine neurons [181]. AK7, another selective SIRT2 inhibitor with a3-benzenesulfonamidophenyl core, increases the levels of acetylated α-tubulin in the striatum of MPTP-treated mice [243]. AK7, also a neuroprotective agent in HD, reduces neurochemical and redox malfunctioning and improves behavioral deficits in MPTP-induced PD mouse models [243, 244]. Treatment with thienopyrimidinone derivatives induces hyperacetylation of α-tubulin via selective SIRT2 inhibition in MCF-7 cell lines [245]. A closely resembling derivative Thieno[3,2-*d*]pyrimidine derivative has been discovered very recently as a novel LRRK2 inhibitor [25], which exerts inhibitory effects on both wild-type and G2019S mutant LRRK2. Understanding its participation in tubulin binding and acetylation could potentially lead to its exploitation as a therapeutic candidate in PD. AK1 significantly restores α-tubulin acetylation dysregulation in sporadic PD cybrids, proving it to be a viable option for treating MT network impairments. Moreover, it improved interactions of MAPs such as physiological tau and α-syn with α-tubulin, generating stabilized MTs, suggesting a protective role in PD [154]. A thioacetylated pentapeptide, YKK(ε-thioAc)AM, is a potent competitive inhibitor of SIRT2, and it increased the acetylation of α-tubulin in PC12 cells. YKK(ε-thioAc)AM also induced a 30% reduction in the enzymatic activity of serum SIRT2 in PD patients. These suggest that YKK(ε-thioAc)AM may be another therapeutic platform for PD by targeting SIRT2 [246]. γ-Mangostin, a derivative from *Garcinia mangostana,* induces a rise of α-tubulin acetylation in MDA-MD-231 and MCF-7 cells via selective and strong inhibition of SIRT2 [247]. γ-Mangostin also shows significant neuroprotective effects in a 6-OHDA-induced PD model by regulating oxidative stress [248]. Additional data proving its α-tubulin acetylation property in PD models exclusively, along with its subsequent impact on motor protein binding and mitochondrial motility, can be fruitful for recognizing SIRT2 inhibitors as a therapeutic line of class for preventing PD progression.

## Conclusion

Despite a decade of studies on α-tubulin K40 acetylation, its role in MT assembly and functioning is still an emerging concept. In addition, the exact neuroprotective effect offered by HDAC6 or SIRT2 inhibitors in neurological diseases is still unclear. Whether the neuroprotection is the result of enhanced α-tubulin acetylation or acetylation of other subcellular targets remains questionable and debatable. Further studies are needed to explore the relationship of HDAC6 and SIRT2 activity with α-tubulin acetylation in neurons. For example, in a most recent study, inhibition of SIRT2 was achieved by transfection of miR-212-5p in SH-SY5Y cells; as a result, enhanced acetylation of p53 was observed but the status of α-tubulin acetylation was not reported. Autophagic flux mediated by acetylated p53 was increased, and neuroprotection of dopaminergic neurons was recorded in vivo [249]. Hence, further studies with robust and accurate findings are required to collectively conclude strong links between α-tubulin acetylation and neuroprotection not only restricted to PD but also in other neurodegenerative conditions. This ‘hidden’ PTM would serve as a game changer in the treatment of neurological or even other diseases displaying a significant lack of acetylated MT. The finding that modulating MT acetylation restores axonal transport of vital organelles across the neuron shows that uncovering additional molecular aspects of tubulin acetylation in neurodegenerative models would pave the way for better therapeutic design and treatment strategies for neurodegenerative diseases.

## Data Availability

Not applicable.

## Abbreviations

MT: Microtubule
PTM: Post-translational modification
PD: Parkinson’s disease
ALS: Amyotrophic lateral sclerosis
α-syn: α-Synuclein
MAP: Microtubule-associated protein
αTAT-1: α-Tubulin acetyl transferase
HDAC6: Histone deacetylase 6
γTuRC: γ–Tubulin ring complex
GTP: Guanosine tri phosphate
SNpc: Substantia nigra pars compacta
BDNF: Brain-derived neurotrophic factor
KIF: Kinesin superfamily protein
TRAK1: Trafficking kinesin-binding protein 1
TRAK2: Trafficking kinesin-binding protein 2
RHOT1: Ras homolog family member T1
RHOT2: Ras homolog family member T2
LRRK2: Leucine-rich repeat kinase 2
PINK1: PTEN-induced kinase 1
BICD2: Bicaudal-D2
DDB: Dynein-dynactin-BICD2
VCP: Valosin-containing protein/p97
Lis1: Lissencephaly1
NDEL1: NudE Neurodevelopment *Protein* 1 Like 1
TPPP/p25: Tubulin Polymerization Promoting Protein
PROTAC: Proteolysis targeting chimeric
TCP: Tubulin Carboxypeptidase
TTL: Tubulin tyrosine ligase
VASH1: Vasohibin-1
SVBP: Small vasohibin-binding protein
KAT: Lysine acetyltransferase
GNAT: Gcn5-related N-acetyltransferases
MPTP: 1-Methyl-4-phenyl-1,2,3,6-tetrahydropyridine
MECP2: Methyl-CpG-binding protein 2
TBA: Tubastatin A
